# The Treatment of Granular Lids

**Published:** 1892-11-19

**Authors:** 


### BATH EYE INFIRMARY.

### The Treatment of Granular Lids.

Many a professional reputation has been lost amidst the granulations of this troublesome disease. Sometimes overlooked, frequently mistaken for simple catarrhal or for other forms of ophthalmia, it provides pitfalls for the careless practitioner on the one hand and a golden harvest for the unscrupulous empiric on the other.

Granular lids, when treated early, may often be cured in a surprisingly short period of time, but if the disease be neglected it runs a chronic course very difficult to cope with, and far-reaching in its disastrous effects. It drags on week after week and month after month till the roughened condition of cornea, called pannus, supervenes, and vision is irreparably impaired. The early symptoms may consist of nothing more than a slight intolerance of light, increased discharge of tears, together with itching and a sensation of a foreign body within the lids. The ocular conjunctiva is seen to be more than usually injected, but it is not until the lower lid is drawn down or the upper lid inverted that the unmistakable sign of granular ophthalmia is manifest. Then there is seen under the conjunctiva, and especially near the *cul de sac*, a number of minute, whitish, semi-transparent spots, resembling millet seeds, and not rising much above the surface of the surrounding inflamed conjunctiva. There is not much secretion at first, but if the case passes on into the chronic stage more is secreted of a muco-purulent character, glueing the lids and intensifying the patient's discomfort. The granulations increase in size and number, the conjunctival papillæ become infiltrated, and the mucous membrane becomes gelatinous. Gradually a fibrinous transformation takes place, followed by cicatricial contraction, and the condition known as trachoma is fully established.

The more serious cases where the follicles are enlarged and cicatricial bands are formed are seldom seen in Bath. Pannus too, as a secondary sequel, seldom occurs there. Mr. Beaumont accounts for this immunity by the hygienic nature of the locality. Situated on the sloping sides of an oolite valley, there is no stagnation of drainage, whilst pure air and fresh water pervade the city of the Sun. There are but few factories, and the children are not congregated in large, ill-ventilated schools, nor are they overcrowded in the houses of the poor. An additional explanation of this immunity may possibly be found also in the fact that there is very little admixture of Celtic blood in the population of Bath. Ireland has been well called the home of trachoma, and the effect of racial tendency towards trachomatous disease is shown by comparing Bath with Exeter. This latter town, with a smaller population than Bath, suffers very considerably from trachoma, and it is well recognised that the population of South Devon and Cornwall is derived originally to a measurable extent from a Celtic stock.

Mr. Beaumont's treatment of granular lids at the Bath Eye Infirmary may be summed up in the word "massage," and is carried out in the following manner. A drop of solution of hydrochlorate of cocaine (gr. x. ad. ℥j) is dropped into the eye three times with an interval of five minutes between each instillation. The upper lid is then inverted in the usual manner and its conjunctiva dusted thickly with finely-powdered boracic acid. The easiest way to accomplish this is to flick it on with a camel's hair brush, which has been previously well charged with the powdered acid, taking care to get it into the *cul de sac*. The puff-balls used so frequently in the application of iodoform to wounds also answer admirably for this purpose. The next step is to rub the powder into the inflamed and granular lid by means of the pulp of the forefinger, in much the same way as a housekeeper rubs salt into raw meat when she wishes to preserve it. The lower lid is then everted and treated similarly. Any remaining superfluity of the powder may be washed away by means of a camel's hair brush moistened with lead lotion. The other eye is then treated in the same way. Some discomfort, usually lasting about half an hour, follows, but it is not often severe or prolonged if the acid has been thoroughly pulverised. The treatment may be repeated twice or three times a week, and after one or two applications there is generally little or no reaction or discomfort; the worse the case is, too, the better does it seem to respond to the remedy. It is often the slight cases that are the most prolonged, but the patient, noting the improvement, readily returns for the completion of his cure. Gradually the intervals between the massage may be lengthened, and finally discontinued. It is surprising how little objection is raised, even by young children, to massage when the lids have been previously cocainised. In some very exceptional cases where the particles of boracic acid dust act as foreign bodies and give rise to discomfort, an ointment may be substituted, consisting of 15 grains of boracic acid in an ounce of lanoline or soft paraffin. This ointment can be rubbed in after the lids have been cocainised, as before described, two or three times a week. For home treatment the patient is ordered a solution of 20 grains of tannin in an ounce of glycerine. This may advantageously be put into one of those oil-tins that are sold for oiling sewing machines, the instructions being that one drop shall be dropped into each eye three times a day. At bed-time a small quantity of ointment, consisting of 8 grains of yellow oxide of mercury to an ounce of white vaseline, may be smeared along the edges of the lids to prevent their sticking, and so causing a retention of the secretion during the night. In addition to the local treatment, the general health, and hygienic measures especially, must be attended to. Internally, cod-liver oil and iron; externally, fresh air and pure water are the lines of treatment that lead to success. All overcrowding, whether in schools, in sleeping apartments, or in factories must be avoided, and separation from healthy companions must be rigourously enforced.

In conclusion, prompt measures and thorough will do much to combat this troublesome malady—patience and perseverance will almost always effect a cure—the restored eyes will reward the successful practitioner and earn for him the gratitude of the patient.

